# Mechanisms underlying neurocognitive dysfunction following critical illness: a systematic review

**DOI:** 10.1111/anae.16494

**Published:** 2024-12-12

**Authors:** Mark Andonovic, Holly Morrison, William Allingham, Robert Adam, Martin Shaw, Tara Quasim, Joanne McPeake, Terence Quinn

**Affiliations:** ^1^ Academic Unit of Anaesthesia, Critical Care and Perioperative Medicine University of Glasgow Glasgow UK; ^2^ Department of Anaesthesia NHS Lanarkshire Glasgow UK; ^3^ Department of Anaesthesia NHS Greater Glasgow and Clyde Glasgow UK; ^4^ The Healthcare Improvement Studies Institute University of Cambridge Cambridge UK; ^5^ School of Cardiovascular and Metabolic Health University of Glasgow Glasgow UK

**Keywords:** intensive care, long‐term outcomes, neurocognitive decline

## Abstract

**Introduction:**

Cognitive impairment is a significant healthcare problem globally and its prevalence is projected to affect over 150 million people worldwide. Survivors of critical illness are impacted frequently by long‐term neurocognitive dysfunction regardless of presenting illness, but the mechanisms are poorly understood. The goal of this review was to synthesise the existing evidence regarding potential mechanisms underlying neurocognitive dysfunction following critical illness in order to guide potential avenues for future research.

**Methods:**

We performed a systematic search of the literature for studies published between 1 January 1974 and 15 July 2023. We included publications involving adult patients with critical illness due to any aetiology that assessed for cognitive impairment following recovery from illness, and explored or investigated potential underlying causative mechanisms. The quality and risk of bias of the individual studies was assessed using the Newcastle‐Ottawa scale.

**Results:**

Of the 7658 reviewed references, 37 studies comprising 4344 patients were selected for inclusion. Most studies were single centre with sample sizes of < 100 patients. The proportion of patients with long‐term cognitive impairment ranged from 13% to 100%. A wide variety of theoretical mechanisms were explored, with biomarkers and neuroimaging utilised most frequently. Many studies reported associations between investigated mechanisms and reduced cognition; several of these mechanisms have been implicated in other forms of long‐term neurodegenerative conditions. Increased levels of inflammatory cytokines during acute illness and white matter hyperintensities on neuroimaging following recovery were the associations reported most commonly.

**Discussion:**

The underlying pathophysiology of neurocognitive decline after critical illness is not yet understood fully. The mechanisms implicated in other neurodegenerative conditions suggest that this may represent an accelerated version of the same processes. Large scale studies are required to further elucidate the cause of this significant problem for survivors of critical illness.

## Introduction

Cognitive impairment represents a significant global burden to those affected, their carers and healthcare systems. Previous work has estimated that 57.4 million people were living with dementia globally when accounting for the severe end of the spectrum of cognitive impairment; this is projected to rise to 152.8 million by 2050 [1]. Manly et al. reported that approximately one‐third of patients aged ≥ 65 y included in a nationally representative study from the USA had mild cognitive impairment or dementia [2]. While this spectrum of disease primarily affected adults aged > 65 y [3], there is a growing recognition that cognitive decline can affect younger adults, with a recent longitudinal UK‐based cohort study suggesting that cognitive decline can be experienced between the ages of 45–49 y [4]. While the prevalence of cognitive impairment in people aged < 65 y is significantly lower [5], a much higher incidence is seen in certain subgroups, including those recovering from critical illness [6, 7].

Post‐intensive care syndrome is recognised increasingly as a phenomenon affecting a significant number of patients following critical illness, with estimates reporting symptoms in > 50% of survivors up to a year following discharge [8, 9]. Post‐intensive care syndrome comprises elements relating to physical, mental, social and cognitive impairments [10, 11]. Pandharipande et al. found that at 1 year following discharge, 26% of patients had global cognition scores similar to patients with Alzheimer's disease [7]. While this highlights the scale of the issue in survivors of critical illness, causative mechanisms have yet to be elucidated. Multiple theoretical mechanisms have been suggested, but to date there is no consensus and most studies have been exploratory in nature. In addition, there is a well‐recognised link between the development of delirium and subsequent long‐term cognitive impairment [12, 13]. Since the reported incidence of delirium in the ICU is up to 87% [14], this may help explain why survivors of critical illness are at heightened risk of long‐term cognitive impairment. Similarly, the underlying mechanisms for this are not fully elucidated.

Given the breadth of postulated mechanisms for this significant clinical problem, we aimed to ascertain the potential mechanisms underlying neurocognitive dysfunction following critical illness and synthesise the findings to identify possible future research avenues.

## Methods

We conducted this review in line with the PRISMA guidelines [15]. We searched Embase, MEDLINE, the Cochrane Central Register of Controlled Trials and Web of Science: Core Collection between 1 January 1974 and 15 July 2023. The search strategy is detailed in online Supporting Information Appendix S1. To identify ongoing trials, additional searches of the International Trials Registry and the National Institutes of Health registry were conducted. Four authors compiled a list of citations from the databases. Upon merging, duplicate citations were automatically removed by the Covidence™ software (Veritas Health Innovation Ltd, Kidlington, UK). The authors then screened titles and abstracts independently against the pre‐defined eligibility criteria. Following abstract and title review, a concluding full text review was conducted. If any conflicts regarding inclusion arose, a fifth author made a final decision on eligibility.

Studies were eligible for inclusion if they involved adult patients (aged ≥ 16 y) with critical illness due to any aetiology who were assessed for cognitive impairment by any method following recovery from illness. Included studies needed to have explored or investigated potential underlying mechanisms and assessments of cognition must have taken place following hospital discharge. There was recognition that investigation of the underlying mechanism would include looking directly for pathological processes and indirect measures such as biomarkers. There were no restrictions on the type of study included. Studies which involved children, patients with primary brain injury (traumatic brain injury or stroke) and review articles were not included. While mechanisms underlying delirium may significantly overlap with long‐term cognitive dysfunction, only articles reporting on long‐term cognitive impairment were included.

Two authors used a pre‐defined form to independently extract key study characteristics and results, including the study population; number of patients within each study; and underlying mechanisms studied. Outcomes extracted from each study included: modality used to assess cognitive impairment; time‐points used for follow‐up; definitions used to define cognitive impairment; number of patients reported to have cognitive impairment; and any observed associations reported by the study. Each study was assessed independently by two authors for potential risk of bias using the Newcastle‐Ottawa scale for observational studies [16]. Studies which failed to report raw data regarding cognitive outcomes and therefore were at risk of reporting bias, were considered when interpreting reported outcomes. To assess mechanisms for potential causal relationships, the Bradford Hill criteria were applied to each study reporting positive associations [17].

Included studies were anticipated to be exploratory and observational with multiple different hypothesised mechanisms; given the variation in reported outcomes, a meta‐analysis was not possible and therefore the data were synthesised in narrative format. The modalities used to identify potential mechanisms were discussed and grouped thematically, based on broad techniques used (such as biomarkers and neuroimaging) and subsets of these methods. Study results were categorised and assessed based on specific aetiologies underlying initial critical illness. Identified lesions on neuroimaging, changes from pre‐illness imaging or identified biomarkers and their reported associations with cognitive outcomes were pooled and discussed. No restrictions were placed on number of studies required for synthesis for investigating individual mechanisms.

## Results

**Figure 1:**
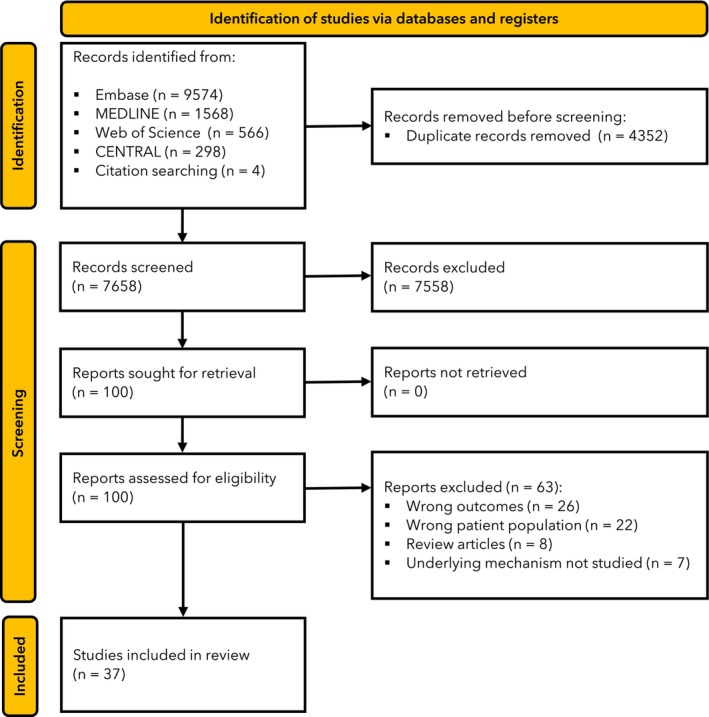
Study flow diagram of studies screened and included in review.

The literature search returned a total 12,010 reports; after automatic duplicate removal, 7658 were retained for title and abstract review of which 100 underwent full text review (Fig. 1). In total, 37 studies which analysed data from 4344 patients were selected for inclusion in this review [18, 19, 20, 21, 22, 23, 24, 25, 26, 27, 28, 29, 30, 31, 32, 33, 34, 35, 36, 37, 38, 39, 40, 41, 42, 43, 44, 45, 46, 47, 48, 49, 50, 51, 52, 53, 54]. The characteristics of the included studies can be found in online Supporting Information Table S1. Study populations ranged in size from one [46] to 794 patients [29]. The selected studies were observational in nature apart from four which performed secondary analyses of randomised controlled trials [23, 45, 48, 52].

The quality assessment for all individual studies selected for inclusion and criteria used to assess them can be found in online Supporting Information Appendix S1 and Table S2; the median (IQR [range]) score was 4 (2–5 [2–9]), with wide variation in the assessed quality of studies selected. Assessment of causal relationships between postulated mechanisms and cognitive impairment can be found in online Supporting Information Tables S3 and S4; all studies met between three and five of the nine criteria used for assessment, demonstrating low to moderate chance of causation.

The included studies reported rates of cognitive impairment diagnosed using several methods. The most common method involved reporting of raw numbers and proportions of patients found to have cognitive impairment; these varied from 13% [47] to 100% [42]. Studies which chose to report cognitive test scores compared with the age‐adjusted normative population scores reported results ranging from ‘borderline’ for cognitive impairment, to below the population mean by 1 SD. Multiple studies, particularly those which compared raw scores on a scale with the investigated underlying mechanism, did not report raw scores or proportions of cognitive impairment. A summary of all results can be found in online Supporting Information Table S5.

We included all critical illness pathologies except for traumatic brain injury or stroke in this review. Among the selected studies, the most common aetiologies represented were respiratory failure, shock or sepsis. Three studies limited inclusion to more severe respiratory failure (either acute lung injury requiring mechanical ventilation [41] or acute respiratory distress syndrome [32, 33]). Furthermore, two studies only included patients treated with respiratory failure requiring extracorporeal membrane oxygenation [31, 53]. While some studies utilised broader inclusion criteria including any patient admitted to ICU, others focused on specific aetiologies. Three of the selected studies included only patients with COVID‐19 [39, 46, 47]; two others investigated cognitive impairment in patients who had an out of hospital cardiac arrest and were subsequently treated with targeted temperature management or therapeutic hypothermia [23, 28].

The investigated mechanisms varied significantly across the 37 selected studies. The proxies used to investigate these potential underlying mechanisms were grouped into four categories: biomarkers; neuroimaging; management strategies and physiological indices during acute illness; and measurement of biological electrical signals. The number of individual studies investigating these are detailed in online Supporting Information Table S6. Studies also varied at the time‐point at which investigations exploring links to long‐term cognitive impairment were conducted. These ranged from point of admission for their critical illness to 17 years after treatment [53] (online Supporting Information Table S1).

The underlying mechanisms investigated most commonly were biomarkers, and were explored in 15 of the selected studies. These studies utilised biomarkers drawn from blood or plasma, but one study also analysed cerebrospinal fluid (CSF) samples collected during ICU admission [44]. Most studies collecting samples for biomarker analysis did so during the acute illness, but one collected at discharge [40] and one 6 weeks after initial presentation [22]. The individual biomarkers investigated varied between studies but included: markers of systemic inflammation (e.g. C‐reactive protein or interleukins); neurobiochemical markers (e.g. S100b protein or neurone specific enolase); biomarkers seen in neurodegenerative conditions (e.g. neurofilament light chains); and single nucleotide polymorphisms associated with dopamine signalling.

Six studies explored markers of systemic inflammation, with the most common being circulating levels of interleukins at various time‐points. Two of these studies identified associations with higher levels of interleukins and worse cognitive impairment [22, 40], whereas the remaining four observed no association [24, 47, 48, 50]. Five studies examined associations including S100b protein and neurone specific enolase, with three finding associations with elevated levels and cognitive impairment [23, 25, 34] and two studies finding no associations [28, 50]. Single studies investigated and found associations between: levels of heparan sulfate fragments and cognitive impairment [20]; single nucleotide polymorphisms and cognitive impairment [18]; and increased plasma amyloid‐β forms and patient self‐reported cognitive impairment [50]. Finally, a single study reported associations between increased CSF levels of soluble TREM2 and neurofilament light chains [44].

Neuroimaging was used frequently across the selected studies with 14 choosing it to explore potential underlying mechanisms [21, 30, 31, 32, 36, 37, 38, 39, 42, 43, 46, 47, 49, 53]. The most common modality (12 studies [21, 30, 31, 36, 38, 39, 42, 43, 46, 47, 49, 53]) was magnetic resonance imaging (MRI), with techniques ranging from diffusion weighted imaging to functional MRI. Fewer studies included patients with computed tomography imaging [32, 36, 47, 49] and a single study chose to employ positron emission tomography (PET) β‐amyloid imaging [37]. Three studies reported associations between white matter hyperintensities or disruptions and cognitive impairment [42, 43, 46]. A single study reported smaller brain volumes at 3 months which were associated with cognitive impairment; these smaller brain volumes were found in the areas typically seen in Alzheimer's disease [30]. However, 10 of the studies did not report any significant associations between neuroimaging abnormalities and cognitive impairment [21, 31, 32, 36, 37, 38, 39, 47, 49, 53]; this included the PET β‐amyloid imaging study used to assess for risk of Alzheimer's disease [37].

Nine studies investigated observed parameters or management strategies employed during critical illness including glycaemic control [27, 33]; blood pressure variability [29, 41]; hypoxaemia [31, 41, 51, 53]; fluid management strategies [41]; and cerebral specific haemodynamic variables [51]. Two studies reported associations between hypoxaemia and cognitive impairment [41, 51]. Conversely, two studies involving patients on extracorporeal membrane oxygenation found no significant associations between hypoxaemia and cognitive impairment [31, 53]. Two further studies reported associations between poor glycaemic control and worse cognitive outcomes [27, 33]. Finally, only one study investigated biological electrical signals consisting of electroencephalogram (EEG) waveform analysis during the period of acute illness [54]; this study reported associations between reduced α power ratio and long‐term cognitive impairment.

## Discussion

**Figure 2:**
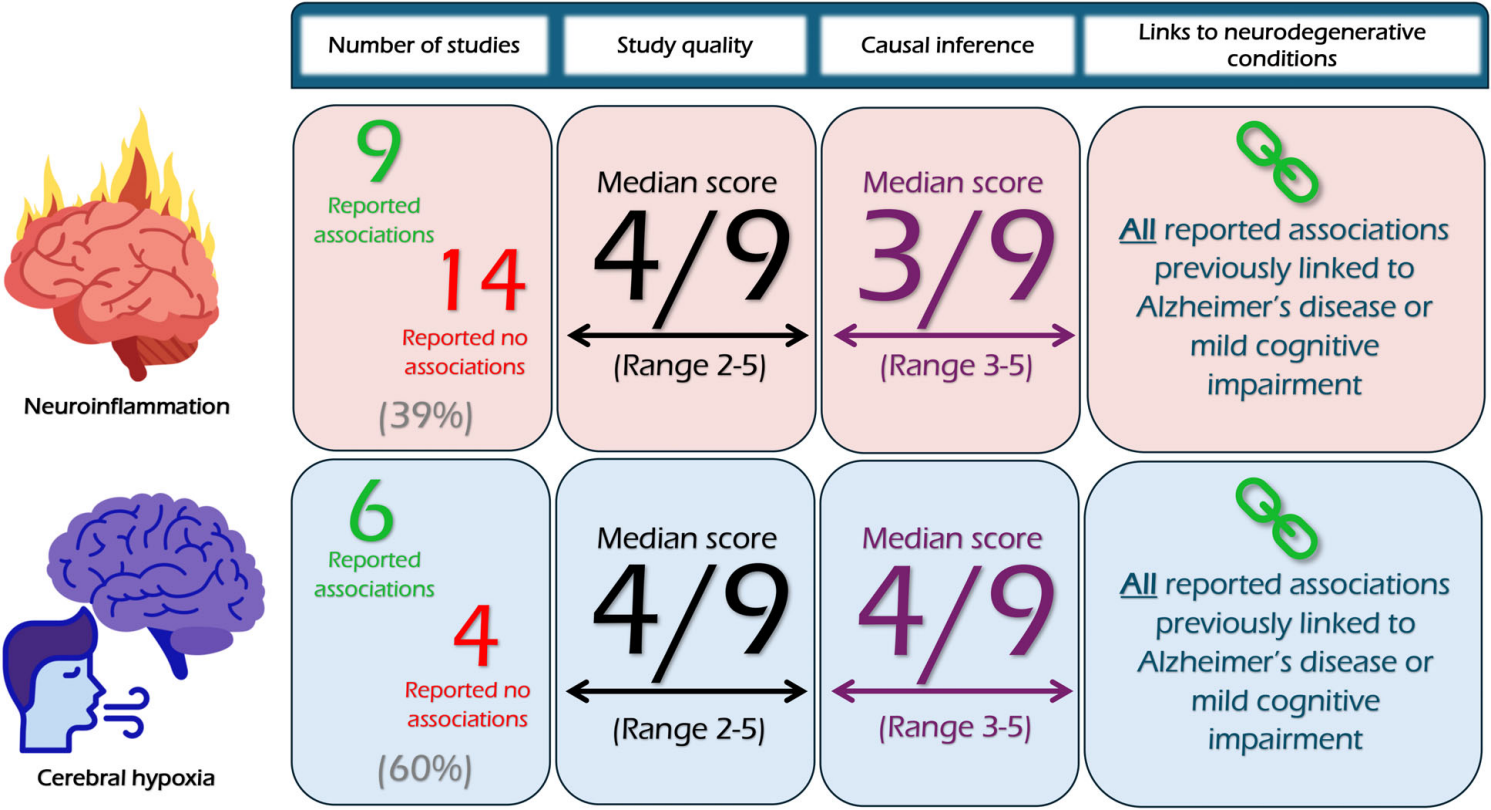
Visual summary of results and data synthesis.

This review revealed a significant number of diverse studies which employed wide‐ranging methodology to explore the mechanisms underlying neurocognitive dysfunction following critical illness. The findings consistently showed that long‐term cognitive impairment is a significant issue in survivors of critical illness. Most of the studies included were exploratory in nature due to the continuing paucity of mechanistic evidence relevant to this field. As a result, many of the 37 studies included in this review had small study populations, with very little overlap in inclusion criteria, explored mechanisms and reported outcomes to allow pooling of data. In addition, most studies were assessed to be poor or fair quality on the Newcastle‐Ottawa scale, which should be considered when interpreting them. Nonetheless, several potential associations described draw links to mechanisms implicated in neurodegenerative conditions such as Alzheimer's disease and mild cognitive impairment. These can be grouped under the broad mechanisms of neuroinflammation and cerebral hypoxia (Fig. 2).

Neuroinflammation is likely in critical illness due to the widespread systemic inflammation frequently seen in patients. Two of the six studies investigating systemic inflammation reported associations with higher levels of interleukins and worse cognitive impairment [22, 40]. A recent systematic review examining inflammation and cognition in older people found that higher levels of inflammatory cytokines (including interleukin‐6) may increase cognitive deterioration [55]. Given how common an acute, severe inflammatory response is in patients who are critically unwell, this observed association is biologically plausible. However, both studies reporting associations were small in size and identified differing cytokines at differing time‐points, so must be interpreted with caution.

Those studies which explored the association between heparan sulfate fragments [20] and single nucleotide polymorphisms [18] were the only studies to investigate these mechanisms and results must be interpreted with caution; the latter study also did not report granular data on raw cognitive scores or rates of impairment, which may limit its interpretation. Nonetheless, higher concentrations of CSF heparan sulfate proteoglycans have been implicated previously in Alzheimer's disease [56]. Similarly, the association between the investigated GSK3B single nucleotide polymorphism has been reported previously as twice as likely to occur in patients with Alzheimer's disease compared with healthy controls [57].

The study reporting associations between increased plasma amyloid‐β forms and cognitive impairment was alone in investigating this mechanism and used self‐reported outcomes which is a limitation [50]. However, this mechanism has also been implicated in long‐term neurodegenerative disorders, although the value of plasma levels is uncertain [58, 59].

White matter hyperintensities and associations with cognitive impairment were reported by three of 14 studies using neuroimaging [42, 43, 46]. However, one was an isolated case report in a patient with COVID‐19 [46] and the other two were small studies conducted in the same centre. White matter hyperintensities are found frequently in other neurodegenerative diseases such as Alzheimer's disease and previous studies have stated associations between regional hyperintensities with the typical amyloid‐β deposition and neurodegeneration of Alzheimer's disease [60].

In the study by Gunther et al., smaller brain volumes at 3 months in the areas typically seen in Alzheimer's disease were associated with worse cognitive impairment [30]. Smaller brain volumes following critical illness have been reported previously, but this earlier study did not assess patients' cognition [61].

Cerebral hypoxia may predominate in critical illness due to either hypoxaemia from the underlying disease process or impaired cerebral autoregulation; this may also be contributed to by cerebral microthrombi depending on the underlying disease process. Five studies examined neurone specific enolase and S100b [23, 25, 28, 34, 50], which are implicated in neuronal cell death, and three reported associations with cognitive impairment. Neurone specific enolase has a more established role in critical care for purposes of neurological prognostication following cardiac arrest [62]. Indeed, one of the selected studies used cardiac arrest as its inclusion criteria [23]. However, the other two studies included patients with sepsis [25] and with respiratory failure or shock [34]. While the role of S100b proteins in Alzheimer's disease pathophysiology is not well understood [63], a recent meta‐analysis demonstrated significantly higher levels of CSF neurone specific enolase in patients with Alzheimer's disease [64].

Two of four studies investigating hypoxaemia stated associations between reduced blood oxygen levels and cognitive impairment [41, 51]. The potential mechanism is likely secondary to reduced cerebral oxygen delivery and utilisation which may lead to oxidative stress, mitochondrial dysfunction and inflammation [65]. In the only study to record EEGs, the authors reported associations with patterns and cognitive impairment [54]. The small sample size limits the conclusions that can be drawn, but α power ratio has previously been reported as correlating with memory impairment in patients with mild cognitive impairment [66].

Certain mechanistic investigations were not linked to either neuroinflammation or cerebral hypoxia. The only study in this review to investigate CSF samples reported associations between increased CSF levels of solubleTREM2 and neurofilament light chains and cognitive impairment, but interpretation of the results is extremely limited by the sample size [44]. Nonetheless, previous work has shown increased levels of neurofilament light chains in CSF are associated with a significant increased risk of mild cognitive impairment [67]. Two studies reported associations between poor glycaemic control and worse cognitive outcomes [27, 33]; there is a well‐recognised link between increased risk of cognitive impairment and poor glycaemic control in the context of diabetes mellitus [68].

The strengths of this review include the robust search strategy employed and adherence to both pre‐planned methodology and data synthesis, which was registered prospectively. However, there are limitations. The significant methodological differences between the studies limited the ability to pool results. Furthermore, due to the exploratory nature of most studies, small sample sizes significantly limit the ability to draw definitive conclusions. This was further limited by some studies not reporting raw cognitive scores or rates of impairment in their study population, which may have resulted in reporting bias of their results. Finally, the quality of the included studies varied significantly, with the majority assessed as being low quality.

In conclusion, despite a wide breadth of studies seeking to understand the mechanisms underpinning critical illness‐related cognitive impairment, the underlying pathophysiology remains poorly understood. Despite the limitations of the studies included, very tentative associations have been reported between neurocognitive decline after critical illness and multiple mechanisms implicated in established neurodegenerative conditions. This may potentially imply an accelerated version of the same process. This review highlights the urgent need for larger, longitudinal studies investigating the underlying causes of this significant problem for survivors of critical illness.

## Supporting information


**Appendix S1.** Finalised search strategy.
**Appendix S2.** Newcastle‐Ottawa criteria applied for quality assessment of individual studies.

**Table S1.** Characteristics of individual studies included in review.
**Table S2.** Quality assessment of studies included in review.
**Table S3.** Bradford Hill criteria applied for causal inference assessment of individual studies.
**Table S4.** Causal inference assessment of individual studies reporting a positive association.
**Table S5.** Reported outcomes of individual studies.
**Table S6.** Proxies used to investigate potential mechanisms underlying cognitive impairment.
