# Supplementary material for: Mechanisms underlying neurocognitive dysfunction following critical illness: a systematic review
Source: Anaesthesia. 2024 Dec 12;80(2):188–96. doi: 10.1111/anae.16494 (PMC11726275; doi:10.1111/anae.16494)
Supplement: Supplementary file 1 — Appendix S1. Finalised search strategy. Appendix S2. Newcastle‐Ottawa criteria applied for quality assessment of individual studies. [file ANAE-80-188-s001.docx]

**Appendix S1**: Finalised search strategy

1. exp Neurocognitive Disorders/
2. Brain/dg, [Diagnostic Imaging, Pathology]
3. Exp Brain Injury, Chronic/
4. ((cogniti* or neurocogniti*) adj5 (disorder* or declin* or dysfunct* or impair* or deficit* or disabilit* or problem* or los* or deteriorat* or degenerat* or complain* or disturb*)).tw.
5. 2 or 4
6. critical illness/
7. Critical Care/
8. Intensive Care Units/
9. (critical* adj (care or ill* or condition)).tw.
10. (lifethreatening illness or life-threatening illness).tw.
11. (intensive care or ICU).tw.
12. “health shock?”.tw.
13. Respiratory Distress Syndrome, Adult/
14. Acute Lung Injury/
15. (ARDS or AHRF).tw.
16. ((acute or adult) adj3 respirator*).tw.
17. (respirat* adj3 (distress or failure)).tw.
18. lung injur*.tw.
19. Respiration, Artificial/
20. ((mechanical* or artificial*) adj3 (ventil* or respirat*)).tw.
21. Sepsis/
22. Shock, Septic/
23. (sepsis or “septic shock”).tw.
24. or/6-23
25. exp Neuroimaging/
26. exp Radiology/
27. Biomarkers/
28. (mechanism or cause or pathophysiology or neuroimaging or imaging or radiology or biomarker or blood).tw.
29. Or/25-28
30. 5 and 24 and 29
31. animal/
32. human/
33. 31 not (31 and 32)
34. 30 not 33

**Appendix S2:** Newcastle Ottawa criteria applied for quality assessment of individual studies

**NEWCASTLE – OTTAWA QUALITY ASSESSMENT SCALE
COHORT STUDIES**

Note: A study can be awarded a maximum of one star for each numbered item within the Selection and Outcome categories. A maximum of two stars can be given for Comparability

**Selection**

1. Representativeness of the exposed cohort
   1. Truly representative of the average critically unwell patient*
   2. Somewhat representative of the average critically unwell patient*
   3. Selected group of users e.g. nurses, volunteers
   4. No description of the derivation of the cohort
2. Selection of the non-exposed cohort
   1. Drawn from the same community as the exposed cohort*
   2. Drawn from a different source
   3. No description of the derivation of the non-exposed cohort
3. Ascertainment of exposure
   1. Secure record (e.g. surgical records)*
   2. Structured interview*
   3. Written self-report
   4. No description
4. Demonstration that outcome of interest was no present at start of study
   1. Yes*
   2. No

**Comparability**

1. Comparability of cohorts on the basis of the design or analysis
   1. Study controls for age*
   2. Study controls for any additional factor*

**Outcome**

1. Assessment of outcome
   1. Independent blind assessment*
   2. Record linkage*
   3. Self-report
   4. No description
2. Was follow-up long enough for outcomes to occur
   1. Yes (cognitive impairment assessed at 3 months or later)*
   2. No
3. Adequacy of follow-up of cohorts
   1. Complete follow-up – all subjects accounted for*
   2. Subjects lost to follow up unlikely to introduce bias/small number lost (>80% follow up, or description provided of those lost)*
   3. Follow up rate <80% and no description of those lost
   4. No statement
