# Supplementary material for: Mechanisms underlying neurocognitive dysfunction following critical illness: a systematic review
Source: Anaesthesia. 2024 Dec 12;80(2):188–96. doi: 10.1111/anae.16494 (PMC11726275; doi:10.1111/anae.16494)
Supplement: Supplementary file 2 — Table S1. Characteristics of individual studies included in review. Table S2. Quality assessment of studies included in review. Table S3. Bradford Hill criteria applied for causal inference assessment of individual studies. Table S4. Causal inference assessment of individual studies reporting a positive association. Table S5. Reported outcomes of individual studies. Table S6. Proxies used to investigate potential mechanisms underlying cognitive impairment. [file ANAE-80-188-s002.docx]

**Table S1:** Characteristics of individual studies included in review

| **Study** | **Design** | **Inclusion criteria** | **Study numbers** | **Underlying mechanism** | **Subset of underlying mechanism** | **Assessed quality of study** |
| --- | --- | --- | --- | --- | --- | --- |
| Amado-Rodriguez et al. [18] | Prospective cohort | Respiratory failure or shock | 321 | Biomarkers (during admission) | Leukocyte DNA single nucleotide polymorphisms involving dopamine type-2 receptor, dysbindin, AKT and GSK3b | 2 |
| Anderson et al. [19] | Prospective cohort | Sepsis + respiratory failure or shock | 50 | Biomarkers (on ICU admission) | Plasma glutamate | 2 |
| Anderson et al. [20] | Prospective cohort | Sepsis + respiratory failure or shock | 20 | Biomarkers (on ICU admission) | Circulating heparan sulfate fragments | 2 |
| Arango-Lasprilla et al. [21] | Prospective cross-sectional | Multi-Organ Dysfunction Syndrome | 26 (13 healthy controls) | Neuroimaging  (timeframe not stated) | MRI (Structural, diffusion tensor and functional at rest) | 3 |
| Arias et al. [22] | Prospective cohort | Sepsis | 64 (11 sepsis, 15 acute non-sepsis illness and 38 healthy controls) | Biomarkers (weeks 0, 1, 2 & 6) | IL-1, IL-4, IL-6, IL-10, and TNFα | 2 |
| Bronnick et al. [23] | RCT (secondary observational analysis) | OOHCA who underwent TTM + Cerebral Performance Score <3 | 79 (36 TTM for 24 hours, 43 TTM for 48 hours) | Biomarkers (admission, 24 hours, 48 hours and 72 hours) | S-100b and NSE | 4 |
| Brummel et al. [24] | Prospective cohort | Respiratory failure or shock | 548 | Biomarkers (day 1, 3 and 5) | CRP, IFN-γ, IL-1β, IL-6, IL-8, IL-10, IL-12, MMP-9, TNFα, soluble TNF receptor 1 and protein C | 5 |
| Calsavara et al. [25] | Prospective cohort | Sepsis | 33 | Biomarkers (at ICU discharge) | NSE and IFN-γ | 4 |
| Cusack et al. [26] | Retrospective cohort | ICU length of stay >48 hours and mechanical ventilation | 27 | Management and indices during acute illness | Receipt of blood transfusion and circadian rhythm disruption | 2 |
| Duning et al. [27] | Retrospective case-control | ICU admission | 74 (37 hypoglycaemic patients with 37 matched controls) | Management and indices during acute illness | Hypoglycaemic episodes | 8 |
| Fugate et al. [28] | Prospective cohort | OOHCA treated with therapeutic hypothermia | 56 | Biomarkers (within 3 days of admission) | NSE | 4 |
| Garbajs et al. [29] | Prospective cohort | ICU patients greater than 50 years old | 794 | Management and indices during acute illness | Blood pressure variability | 6 |
| Gunther et al. [30] | Prospective cohort | Respiratory failure or shock | 47 | Neuroimaging (at 3 months) | MRI (total brain volume and ventricle to brain ratio) | 5 |
| Holzgraefe et al. [31] | Prospective cohort | Patients with H1N1 influenza treated with ECMO | 7 | Management and indices during acute illness and neuroimaging (at follow up) | Hypoxaemia during ECMO and MRI (structural) | 5 |
| Hopkins et al. [32] | Prospective cohort | ARDS | 30 (15 ARDS and 15 matched controls) | Neuroimaging (within 40 days of ARDS diagnosis) | CT (brain volumes) | 7 |
| Hopkins et al. [33] | Retrospective cohort | ARDS | 66 | Management and indices during acute illness | Glycaemic control | 5 |
| Hughes et al. [34] | Prospective cohort | Respiratory failure or shock | 419 | Biomarkers (within 72 hours of admission) | E-selectin, PAI-1, S100b, UCHL1 and BDNF | 5 |
| Hughes et al. [35] | Prospective cohort | Respiratory failure or shock | 272 | Biomarkers (day 1, 3, 5 and 7) | Plasma acetylcholinesterase and butyrylcholinesterase | 4 |
| Jackson et al. [36] | Prospective cohort | Respiratory failure or shock | 47 | Neuroimaging (at 3 months) | Functional MRI | 5 |
| Jackson et al. [37] | Prospective case series | Respiratory failure or shock | 14 | Neuroimaging (between 2-4 years after discharge) | Florbetapir-PET β amyloid imaging | 5 |
| Jimenez-Marin et al. [38] | Prospective cross-sectional | Multi-organ failure | 44 (22 multi-organ failure and 22 healthy controls) | Neuroimaging (at 6 months) | MRI (structural, T1 and functional BOLD) | 8 |
| Klinkhammer et al. [39] | Prospective cohort | COVID-19 | 205 (101 ICU and 104 general hospital) | Neuroimaging (at least 6 months following discharge) | MRI (T1 and T2 weighted and diffusion weighted imaging) | 6 |
| Maciel et al. [40] | Prospective cohort | ICU patients with at least one episode of delirium | 60 | Biomarkers (at hospital discharge) | IL-6 and IL-10 | 4 |
| Mikkelsen et al. [41] | Prospective cohort | Mechanically ventilated patients with acute lung injury | 122 | Management and indices during acute illness | Fluid management strategy, haemodynamics and hypoxaemia | 2 |
| Morandi et al. [42] | Prospective case series | Respiratory failure or shock with documented delirium | 8 | Neuroimaging (during ICU admission) | MRI (structural) | 4 |
| Morandi et al. [43] | Prospective cohort | Respiratory failure or shock with documented delirium | 47 | Neuroimaging (during ICU admission) | MRI (structural and diffusion tensor imaging) | 5 |
| Orhun et al. [44] | Prospective cohort | Sepsis associated encephalopathy | 11 | Biomarkers (during ICU admission) | Serum sTREM2 and cerebrospinal fluid sTREM2 and NFL | 6 |
| Page et al. [45] | RCT (secondary observational analysis) | Mechanically ventilated ICU patients | 142 | Biomarkers (day 1, 3, 7, 14 and 28) | Plasma NFL | 3 |
| Petersson et al. [46] | Case report | COVID-19 | 1 | Neuroimaging (at 7 months) | MRI (T1 and T2, T2-FLAIR, diffusion weighted imaging and susceptibility weighted imaging) | 3 |
| Ragheb et al. [47] | Prospective cohort | COVID-19 | 148 | Neuroimaging and biomarkers (during admission) | MRI or CT, WBC, CRP, Ferritin, LDH, IL-6 and D-dimer | 2 |
| Serrano et al. [48] | RCT (secondary observational analysis) | ICU patients with at least one episode of delirium | 251 | Biomarkers (day 1, day 8 or at discharge) | CRP, IL-6, IL-8, IL-10 and TNFα | 2 |
| Suchyta et al. [49] | Retrospective cohort | All ICU patients with available neuroimaging | 64 | Neuroimaging (during admission) | MRI or CT | 2 |
| Van den Boogaard et al. [50] | Prospective cohort | ICU patients screened for delirium during admission | 100 | Biomarkers (during admission) | TNFα, IL-1β, IL-6, IL-8, IL-17, IL-18, MIF, IL-1RA, IL-10, MCP-1, HNP-1, CRP, PCT, Amyloidβ1-42, 1-40 and S100b | 5 |
| Victor et al. [51] | Prospective cohort | Mechanically ventilated patients assessed for Post-intensive care syndrome | 4 | Management and indices during acute illness | Cerebral haemodynamic variables (CaO2, DO2, ICP, CPP), PEEP changes, average speed in MCA and average speed in OA | 2 |
| Vitamin Improve Outcomes Leveragin et al. [52] | RCT (secondary observational analysis) | ICU patients with one or more risk factor for ARDS and vitamin D deficiency | 95 (47 in vitamin D3 treatment group and 48 in placebo) | Management and indices during acute illness | Vitamin D supplementation | 4 |
| Von Bahr et al. [53] | Prospective cohort | ECMO patients | 38 | Management and indices during acute illness  and neuroimaging (at follow up) | Hypoxaemia whilst on ECMO and MRI (structural) | 2 |
| Williams Roberson et al. [54] | Prospective cohort | Respiratory failure or shock | 10 | Biological electrical signals (during admission) | EEG | 4 |

ARDS, acute respiratory distress syndrome; BDNF, brain derived neurotrophic factor; BOLD, blood oxygen level dependent; CaO2, arterial content of oxygen; CPP, cerebral perfusion pressure; CRP, C-reactive protein; CT, computed tomography; DNA, deoxyribose nucleic acid; DO2, delivery of oxygen; ECMO, extra corporeal membrane oxygenation; EEG, electroencephalogram; FLAIR, fluid attenuated inversion recovery; GSK3b, glycogen synthase kinase 3 beta; HNP-1, human neutrophil peptide-1; ICP, intracranial pressure; ICU, intensive care unit; IFN, interferon; IL, interleukin; LDH, lactate dehydrogenase; MCA, middle cerebral artery; MCP-1, monocyte chemoattractant protein-1; MIF, macrophage migration inhibitory factor; MMP-9, matrix metalloproteinase 9; MRI, magnetic resonance imaging; NFL, neurofilament light chain; NSE, neuron specific enolase; OA, occipital artery; OOHCA, out of hospital cardiac arrest; PAI-1, plasminogen activator inhibitor-1; PCT, procalcitonin; PEEP, positive end expiratory pressure; PET, positron emission tomography; RCT, randomised controlled trial; S100b, S100 calcium binding protein B; sTREM2, soluble triggering receptor expressed on myeloid cells 2; TNFα, tumour necrosis factor alpha; TTM, targeted temperature management; UCHL1, ubiquitin carboxy-terminal hydrolase L1; WBC, white blood cells

**Table S2:** Quality assessment of studies included in review

| **Study** | **Year** | **Selection (max 4)** | **Comparability (max 2)** | **Outcome (max 3)** | **Total (max 9)** |
| --- | --- | --- | --- | --- | --- |
| Amado-Rodriguez et al. | 2018 | 1 | 0 | 1 | **2** |
| Anderson et al. | 2017 | 2 | 0 | 0 | **2** |
| Anderson et al. | 2018 | 1 | 0 | 1 | **2** |
| Arango-Lasprilla et al. | 2019 | 0 | 2 | 1 | **3** |
| Arias et al. | 2017 | 1 | 0 | 1 | **2** |
| Bronnick et al. | 2021 | 1 | 0 | 3 | **4** |
| Brummel et al. | 2021 | 2 | 0 | 3 | **5** |
| Calsavara et al. | 2018 | 2 | 0 | 2 | **4** |
| Cusack et al. | 2019 | 1 | 0 | 1 | **2** |
| Duning et al. | 2010 | 3 | 2 | 3 | **8** |
| Fugate et al. | 2013 | 1 | 0 | 3 | **4** |
| Garbajs et al. | 2022 | 3 | 0 | 3 | **6** |
| Gunther et al. | 2012 | 2 | 0 | 3 | **5** |
| Holzgraefe et al. | 2017 | 2 | 0 | 3 | **5** |
| Hopkins et al. | 2006 | 2 | 2 | 3 | **7** |
| Hopkins et al. | 2010 | 2 | 0 | 3 | **5** |
| Hughes et al. | 2018 | 2 | 0 | 3 | **5** |
| Hughes et al. | 2022 | 2 | 0 | 2 | **4** |
| Jackson et al. | 2015 | 2 | 0 | 3 | **5** |
| Jackson et al. | 2018 | 2 | 0 | 3 | **5** |
| Jimenez-Marin et al. | 2020 | 4 | 2 | 2 | **8** |
| Klinkhammer et al. | 2023 | 3 | 0 | 3 | **6** |
| Maciel et al. | 2019 | 2 | 0 | 2 | **4** |
| Mikkelsen et al. | 2012 | 1 | 0 | 1 | **2** |
| Morandi et al. | 2010 | 2 | 0 | 2 | **4** |
| Morandi et al. | 2012 | 2 | 0 | 3 | **5** |
| Orhun et al. | 2021 | 2 | 2 | 2 | **6** |
| Page et al. | 2022 | 2 | 0 | 1 | **3** |
| Petersson et al. | 2022 | 1 | 0 | 2 | **3** |
| Ragheb et al. | 2021 | 1 | 0 | 1 | **2** |
| Serrano et al. | 2019 | 0 | 0 | 2 | **2** |
| Suchyta et al. | 2010 | 1 | 0 | 1 | **2** |
| Van den Boogaard et al. | 2011 | 2 | 1 | 2 | **5** |
| Victor et al. | 2019 | 1 | 0 | 1 | **2** |
| Vitamin Improve Outcomes Leveragin et al. | 2021 | 2 | 0 | 2 | **4** |
| Von Bahr et al. | 2018 | 1 | 0 | 1 | **2** |
| Williams Roberson et al. | 2022 | 3 | 0 | 1 | **4** |

**Table S3:** Bradford Hill criteria applied for causal inference assessment of individual studies

| **Bradford Hill criteria** | **Description** |
| --- | --- |
| Strength | Study reporting statistically significant relative risk of >1 in critically ill population compared with controls |
| Consistency | More than one included study reporting an association between mechanism and cognitive impairment |
| Specificity | Investigated mechanism specific to the critically unwell population |
| Temporality | Cognitive impairment occurs after the proposed mechanism |
| Gradient | Study reports worsening rates or severity of cognitive impairment with worsening severity of critical illness |
| Plausibility | Biologically plausible link between proposed mechanism and cognitive impairment |
| Coherence | Previous reported data linking the investigated mechanism to cognitive impairment in the critically unwell |
| Experiment | Previous experimental studies in critically unwell related to measures employed to reduce exposure to the proposed mechanism |
| Analogy | Analogous relationship between proposed mechanism and other long-term neurodegenerative conditions |

**Table S4:** Causal inference assessment of individual studies reporting a positive association

| **Study** | **Year** | **Strength** | **Consistency** | **Specificity** | **Temporality** | **Gradient** | **Plausibility** | **Coherence** | **Experiment** | **Analogy** |
| --- | --- | --- | --- | --- | --- | --- | --- | --- | --- | --- |
| Amado-Rodriguez et al. | 2018 | N | N | N | Y | N | Y | N | N | Y |
| Anderson et al. | 2017 | N | N | N | Y | N | Y | N | N | Y |
| Anderson et al. | 2018 | N | N | N | Y | N | Y | N | N | Y |
| Arias et al. | 2017 | N | N | N | Y | N | Y | N | N | Y |
| Bronnick et al. | 2021 | N | Y | N | Y | N | Y | N | Y | Y |
| Calsavara et al. | 2018 | N | Y | N | Y | N | Y | N | Y | Y |
| Cusack et al. | 2019 | N | N | N | Y | N | Y | N | N | Y |
| Duning et al. | 2010 | N | Y | N | Y | N | Y | N | Y | Y |
| Gunther et al. | 2012 | N | N | N | Y | N | Y | N | N | Y |
| Hopkins et al. | 2010 | N | Y | N | Y | N | Y | N | Y | Y |
| Hughes et al. | 2018 | N | N | N | Y | N | Y | N | N | Y |
| Jimenez-Marin et al. | 2020 | N | N | N | Y | N | Y | N | N | Y |
| Klinkhammer et al. | 2023 | N | N | N | Y | N | Y | N | N | Y |
| Maciel et al. | 2019 | N | N | N | Y | N | Y | N | N | Y |
| Mikkelsen et al. | 2012 | N | Y | N | Y | N | Y | N | Y | Y |
| Morandi et al. | 2010 | N | N | N | Y | N | Y | N | N | Y |
| Morandi et al. | 2012 | N | N | N | Y | N | Y | N | N | Y |
| Orhun et al. | 2021 | N | N | N | Y | N | Y | N | N | Y |
| Petersson et al. | 2022 | N | N | N | Y | N | Y | N | N | Y |
| Van den Boogaard et al. | 2011 | N | N | N | Y | N | Y | N | N | Y |
| Victor et al. | 2019 | N | Y | N | Y | N | Y | N | Y | Y |
| Williams Roberson et al. | 2022 | N | N | N | Y | N | Y | N | N | Y |

**Table S5:** Reported outcomes of individual studies

| **Study** | **Cognitive tests** | **Follow-up timepoints** | **Definition of cognitive impairment** | **Cognitive outcomes** | **Associations** |
| --- | --- | --- | --- | --- | --- |
| Amado-Rodriguez et al. [18] | RBANS and TMT-B | 3 and 12 months | Raw scores compared on scale to single nucleotide polymorphisms | Raw scores or proportion of cognitive impairment not reported | Single nucleotide polymorphisms affecting dopamine signalling significantly associated with cognitive impairment |
| Anderson et al. [19] | MoCA and TMT-B | Hospital discharge | MoCA <21 | 9 patients out of 25 survivors assessed (36%) reported to have cognitive impairment | Higher glutamate concentrations associated with cognitive impairment |
| Anderson et al. [20] | MoCA | Hospital discharge | MoCA score <19 | 6 patients (30%) reported to have cognitive impairment | Higher early concentrations of fragments associated with cognitive impairment |
| Arango-Lasprilla et al. [21] | Not stated | Not stated | Not stated | Not stated | No difference in cognitive impairment between multi-organ dysfunction syndrome patients and healthy controls |
| Arias et al. [22] | MMSE | Weeks 0, 1, 2 & 6 | Not stated | Not stated | IL-1 and IL-4 at week 0 negatively correlated and TNFα positively correlated with MMSE scores in sepsis patients |
| Bronnick et al. [23] | RAVLT, ROCFT, WAIS-IV, TMT-A, TMT-B and DKEFS Verbal Fluency | 6 months | z-scores <-1.66 | 17 patients (22%) reported to have cognitive impairment | Higher levels of neuron-specific enolase at 48 hours associated with cognitive impairment |
| Brummel et al. [24] | RBANS and TMT-B | 3 and 12 months | Scores compared to age adjusted mean | Scores approximately 1 standard deviation below age-adjusted population means | No association observed between biomarkers and cognitive scores |
| Calsavara et al. [25] | CERAD Battery total score, MMSE, TMT-A and TMT-B | ICU discharge and 12 months | Inability to complete TMT-A or TMT-B in under 300 seconds | 11 patients (69%) – only 16 assessed at 1 year | Increased neuron-specific enolase and IFN-γ associated with worse cognitive impairment |
| Cusack et al. [26] | Mental Component Health Related Quality of Life | 6 months | Scores below the population average | 11 patients (41%) reported to have cognitive impairment | Higher rate of transfusions and circadian rhythm disruption associated with cognitive impairment |
| Duning et al. [27] | MMSE, TMT-A, TMT-B, ROCFT, RWFT and WMS | At least 1 year post discharge | Between group difference in scores | Average scores in both groups below population average for most domains | Episodes of hypoglycaemia associated with worse visuospatial skills |
| Fugate et al. [28] | TICS-m | Range from 2-59 months | TICS-m score <32 | 22 patients (40%) reported to have cognitive impairment | No significant associations with cognitive impairment reported |
| Garbajs et al. [29] | TMT-B, BNT, WMS and WAIS | At least 6 months post discharge | Change in z-score from pre-illness cognitive testing | The 371 patients assessed for long-term cognition had accelerated decline | No association between blood pressure variability and long-term cognitive trajectory |
| Gunther et al. [30] | RBANS, TMT-A and TMT-B | 3 months and 12 months | Raw scores compared on scale to MRI indices | Mean RBANS score of study population classified as borderline | Smaller brain volumes at 3 months associated with long-term cognitive impairment |
| Holzgraefe et al. [31] | Full scale intelligence quotient calculated from WAIS-IV, RAVLT and ROCFT | 3 years | Global cognitive score compared to normal values | One patient (14%) below 1.5 SD of population normal score | Hypoxaemia on ECMO not associated with bad cognitive outcome |
| Hopkins et al. [32] | WAIS, WMS, RAVLT, ROCFT, TMT-A and TMT-B | 12 months | Raw cognitive scores | All ARDS patients had significant memory impairments | No association between brain atrophy and cognitive impairment |
| Hopkins et al. [33] | WAIS, WMS, RAVLT, ROCFT, TMT-A, TMT-B and VFT | 12 months | Two or more test scores >1.5 or one test score >2 standard deviations below the age corrected population mean | 30 patients (46%) reported to have cognitive impairment | Blood glucose dysregulation (moderate hyperglycaemia) associated with worse cognitive outcomes |
| Hughes et al. [34] | RBANS and TBT-B | 3 months and 12 months | Raw global cognition scores | Mean scores for study population below age adjusted population scores | Higher levels S100b and E-selectin associated with long-term cognitive impairment |
| Hughes et al. [35] | RBANS, TMT-B and TICS | 3-6 months | Two or more test scores >1.5 or one test score >2 standard deviations below the age corrected population mean | 72 patients (47%) reported to have cognitive impairment | No associations found between cholinesterase activity and long-term cognitive impairment |
| Jackson et al. [36] | RBANS, TMT-A and TMT-B | 3 months and 12 months | Raw global cognition scores | Scores or proportion of cognitive impairment not stated | No association between fMRI activation patterns and cognitive outcomes |
| Jackson et al. [37] | RBANS | 3 months, 1 year, 4 years and 6 years | Change in >6 points in RBANS score between first and final assessment | 6 patients (43%) with cognitive impairment | No association between amyloid accumulation and cognitive impairment |
| Jimenez-Marin et al. [38] | ROCFT, VFT, HVLT-R, TMT-A, TMT-B, SDMT and BNT | 6 months | Raw scores compared with healthy controls | No significant differences in any domains between patients and healthy controls | Default mode network hyperconnectivity associated with worse attention |
| Klinkhammer et al. [39] | MoCA, TMT-A, TMT-B, Stroop, COWAT, RAVLT and BNT | At least 6 months after discharge | MoCA <26 or z-score <-2.0 for each other test | 86 patients (42%) with cognitive dysfunction | Increased microbleeds in ICU patients but not associated with worse cognitive outcomes compared with non-ICU patients |
| Maciel et al. [40] | MMSE | Between 20 months and 64 months after discharge | MMSE <24 points | 14 patients (23%) with cognitive impairment | Higher levels of IL-6 and IL-10 at hospital discharge associated with long-term cognitive impairment |
| Mikkelsen et al. [41] | WMS-III, WAIS-III, NCSE, HSCT and COWAT | 2 months and 12 months | Score in one domain >2 SD below the age-adjusted population normative data | 41 patients out of 75 assessed at 12 months (55%) with cognitive impairment | Hypoxaemia during acute illness associated with long-term cognitive impairment |
| Morandi et al. [42] | RBANS and TMT-B | 3 months | Raw scores compared to age and education normative scores | All patients reported to have severe impairments | White matter hyperintensities seen in majority (75%) of patients, all of whom had severe cognitive impairment |
| Morandi et al. [43] | RBANS and TMT-B | 3 months and 12 months | Raw scores compared on scale to MRI indices | Mean RBANS score of study population classified as borderline | White matter disruption associated with worse cognitive scores at 12 months |
| Orhun et al. [44] | MMSE and ACE-R | 1 week, 3 months and 12 months | MMSE <18 and ACE-R <82 | 2 out of 4 patients assessed at 3 months found to have cognitive impairment | Elevated cerebrospinal fluid sTREM2 and NFL levels associated with cognitive impairment |
| Page et al. [45] | BTACT | 6 months | Raw scores compared on scale to NFL levels | Scores or proportion of cognitive impairment not stated | No association between NFL measures and cognitive scores |
| Petersson et al. [46] | Not stated | 10 months | Not stated | Impairment in auditory verbal learning, memory recovery, executive function, and attention | White matter hyperintensities and cerebral microbleeds or microthrombi seen in patient with long-term cognitive impairment |
| Ragheb et al. [47] | Cognitive Function Abilities 4a and SBT | Between 1-6 months after discharge | SBT score >4 | 3 out of 22 patients assessed (13%) reported to have cognitive Impairment | No neuroimaging or biomarkers associations with long-term cognitive outcomes |
| Serrano et al. [48] | Surrogate markers using prescription of drugs used to treat Alzheimer’s Disease | Up to 2 years after discharge | Any prescription of Memantine or anti-cholinesterase inhibitors | Not stated | No statistically significant associations between biomarkers and cognitive impairment seen |
| Suchyta et al. [49] | Not stated | At discharge | Not stated | 22 out of 46 survivors (48%) reported to have cognitive impairment | No association between neuroimaging and long-term cognitive impairment |
| Van den Boogaard et al. [50] | CFQ | 18 months (median follow up) | Raw scores compared to biomarkers profiles | Raw scores or proportion of cognitive impairment not reported | Increased ratio of amyloid β proteins associated with reduced long-term cognitive scores |
| Victor et al. [51] | MoCA | 1 month | Not stated | 3 (75%) of patients stated to have reduced cognitive scores | Suggested association between reduced DO2 and reduced cognitive scores |
| Vitamin Improve Outcomes Leveragin et al. [52] | RBANS and DKEFS | At least 8 months after enrolment | Raw scores compared to population means | Adjusted RBANS score for study population less than population mean | Vitamin-D supplementation in deficient patients was not associated with changes in cognitive outcomes |
| Von Bahr et al. [53] | Full scale intelligence quotient and memory index calculated from WAIS-IV, RAVLT, ROCFT, WMS-III, DKEFS | Between 3 and 17 years after treatment | Raw scores compared with age-matched healthy population scores | 6 patients below 1 SD scores for full scale intelligence quotient | Hypoxaemia on ECMO not associated with long-term cognitive dysfunction |
| Williams Roberson et al. [54] | RBANS | 12 months | Raw scores compared to age and education normative scores | All patients demonstrated cognitive impairment in at least one domain | General background slowing, relative alpha power and theta-range spectral variability associated with impairment in certain cognitive domains |

ACE-R, Addenbrooke’s cognitive examination – revised; BNT, Boston naming test; BTACT, brief test of adult cognition by telephone; CERAD, consortium to establish a registry for Alzheimer’s disease; CFQ, cognitive failures questionnaire; COWAT, controlled oral word association test; DKEFS, Delis-Kaplan executive function system; DO2, delivery of oxygen; ECMO, extra corporeal membrane oxygenation; fMRI, functional magnetic resonance imaging; HSCT, Hayling sentence completion test; HVLT-R, Hopkins verbal learning test – revised; ICU, intensive care unit; IFN, interferon; IL, interleukin; MMSE, mini mental state examination; MoCA, Montreal cognitive assessment; MRI, magnetic resonance imaging; NCSE, neurobehavioral cognitive status examination; NFL, neurofilament light chain; RALVT, Rey auditory verbal learning test; RBANS, repeatable battery for the assessment of neuropsychological status; ROCFT, Rey Osterrieth complex figure test; RWFT, Regensburg word fluency test; S100b, S100 calcium binding protein B; SBT, short blessed test; SD, standard deviation; SDMT, symbol digit modalities test; sTREM2, soluble triggering receptor expressed on myeloid cells 2; TICS, telephone interview for cognitive status; TICS-m, modified telephone interview for cognitive status; TMT-A, Trail making test A; TMT-B, Trail making test B; TNFα, tumour necrosis factor alpha; VFT, verbal fluency test; WAIS, Wechsler adult intelligence scale; WMS, Wechsler memory scale;

**Table S6:** Proxies used to investigate potential mechanisms underlying cognitive impairment

| **Underlying mechanism** | **Subset of underlying mechanism** | **Number of studies** |
| --- | --- | --- |
| Biomarkers – analysed from blood samples unless stated otherwise  (15 studies total) | Amyloidβ1-42 and 1-40 | 1 study |
|  | BDNF | 1 study |
|  | Cholinesterases (acetyl- and butyrylcholinesterase) | 1 study |
|  | CRP | 4 studies |
|  | D-dimer | 1 study |
|  | E-selectin | 1 study |
|  | Ferritin | 1 study |
|  | Glutamate | 1 study |
|  | Heparan sulfate fragments | 1 study |
|  | HNP-1 | 1 study |
|  | IFN-γ | 2 studies |
|  | IL-1 | 1 study |
|  | IL-1β | 2 studies |
|  | IL-1RA | 1 study |
|  | IL-4 | 1 study |
|  | IL-6 | 6 studies |
|  | IL-10 | 5 studies |
|  | IL-12 | 1 study |
|  | IL-17 | 1 study |
|  | IL-18 | 1 study |
|  | LDH | 1 study |
|  | MCP-1 | 1 study |
|  | MIF | 1 study |
|  | MMP-9 | 1 study |
|  | NFL (cerebrospinal fluid) | 1 study |
|  | NFL (plasma) | 1 study |
|  | NSE | 3 studies |
|  | PAI-1 | 1 study |
|  | PCT | 1 study |
|  | Protein C | 1 study |
|  | S100b | 2 studies |
|  | Single nucleotide polymorphisms | 1 study |
|  | sTREM2 (cerebrospinal fluid and plasma) | 1 study |
|  | Soluble TNF receptor 1 | 1 study |
|  | TNFα | 4 studies |
|  | UCHL1 | 1 study |
|  | WBC | 1 study |
| Neuroimaging  (14 studies total) | CT | 3 studies |
|  | Florbetapir-PET β amyloid imaging | 1 study |
|  | MRI (diffusion tensor imagingl) | 2 studies |
|  | MRI (diffusion weighted imaging) | 2 studies |
|  | MRI (functional) | 2 studies |
|  | MRI (functional BOLD) | 1 study |
|  | MRI (structural) | 11 studies |
|  | MRI (T1 weighted) | 3 studies |
|  | MRI (T2 weighted and T2-FLAIR) | 2 studies |
| Management and indices during acute illness  (9 studies total) | Blood pressure variability | 1 study |
|  | Cerebral haemodynamic variables (CaO2, DO2, ICP, CPP) | 1 study |
|  | Cerebral artery velocities (MCA and OA) | 1 study |
|  | Circadian rhythm disruption | 1 study |
|  | Fluid management strategy | 1 study |
|  | Glycaemic control | 2 studies |
|  | Haemodynamics | 1 study |
|  | Hypoxaemia | 4 studies |
|  | PEEP changes | 1 study |
|  | Receipt of blood transfusion | 1 study |
|  | Vitamin D supplementation (in deficient individuals) | 1 study |
| Biological electrical signals  (1 study total) | EEG | 1 study |

BDNF, brain derived neurotrophic factor; BOLD, blood oxygen level dependent; CaO2, arterial content of oxygen; CPP, cerebral perfusion pressure; CRP, C-reactive protein; CT, computed tomography; DO2, delivery of oxygen; EEG, electroencephalogram; FLAIR, fluid attenuated inversion recovery; HNP-1, human neutrophil peptide-1; ICP, intracranial pressure; IFN, interferon; IL, interleukin; LDH, lactate dehydrogenase; MCA, middle cerebral artery; MCP-1, monocyte chemoattractant protein-1; MIF, macrophage migration inhibitory factor; MMP-9, matrix metalloproteinase 9; MRI, magnetic resonance imaging; NFL, neurofilament light chain; NSE, neuron specific enolase; OA, occipital artery; PAI-1, plasminogen activator inhibitor-1; PCT, procalcitonin; PEEP, positive end expiratory pressure; PET, positron emission tomography; S100b, S100 calcium binding protein B; sTREM2, soluble triggering receptor expressed on myeloid cells 2; TNFα, tumour necrosis factor alpha; UCHL1, ubiquitin carboxy-terminal hydrolase L1; WBC, white blood cells
